# Potential Molecular Target Prediction and Docking Verification of Hua-Feng-Dan in Stroke Based on Network Pharmacology

**DOI:** 10.1155/2020/8872593

**Published:** 2020-10-28

**Authors:** Ping Yang, Haifeng He, Shangfu Xu, Ping Liu, Xinyu Bai

**Affiliations:** ^1^Key Laboratory of Basic Pharmacology of Ministry of Education and Joint International Research Laboratory of Ethnomedicine of Ministry of Education, Zunyi Medical University, Zunyi 563000, China; ^2^Department of Clinical Pharmacy, Key Laboratory of Basic Pharmacology of Guizhou Province and School of Pharmacy, Zunyi Medical University, Zunyi, Guizhou 563000, China

## Abstract

**Objective:**

Hua-Feng-Dan (HFD) is a Chinese medicine for stroke. This study is to predict and verify potential molecular targets and pathways of HFD against stroke using network pharmacology.

**Methods:**

The TCMSP database and TCMID were used to search for the active ingredients of HFD, and GeneCards and DrugBank databases were used to search for stroke-related target genes to construct the “component-target-disease” by Cytoscape 3.7.1, which was further filtered by MCODE to build a core network. The STRING database was used to obtain interrelationships by topology and to construct a protein-protein interaction network. GO and KEGG were carried out through DAVID Bioinformatics. Autodock 4.2 was used for molecular docking. BaseSpace was used to correlate target genes with the GEO database.

**Results:**

Based on OB ≥ 30% and DL ≥ 0.18, 42 active ingredients were extracted from HFD, and 107 associated targets were obtained. PPI network and Cytoscape analysis identified 22 key targets. GO analysis suggested 51 cellular biological processes, and KEGG suggested that 60 pathways were related to the antistroke mechanism of HFD, with p53, PI3K-Akt, and apoptosis signaling pathways being most important for HFD effects. Molecular docking verified interactions between the core target (CASP8, CASP9, MDM2, CYCS, RELA, and CCND1) and the active ingredients (beta-sitosterol, luteolin, baicalein, and wogonin). The identified gene targets were highly correlated with the GEO biosets, and the stroke-protection effects of Xuesaitong in the database were verified by identified targets.

**Conclusion:**

HFD could regulate the symptoms of stroke through signaling pathways with core targets. This work provided a bioinformatic method to clarify the antistroke mechanism of HFD, and the identified core targets could be valuable to evaluate the antistroke effects of traditional Chinese medicines.

## 1. Introduction

Hua-Feng-Dan (HFD) is a classical Chinese medicine preparation for the treatment of neurological disorders since the Ming dynasty. After more than 370 years of historical inheritance, it is listed as the National Protection Heritage in 1950 and is still in clinical use today. HFD consists of *Aconitum coreanum* (Bai Fu Zi), *Arum ternatum* Thunb. (Ban Xia), *Arisaema erubescens* (Tian Nan Xing), Aconiti Radix (Chuan Wu), Curcumae Radix (Yu Jin), *Gastrodia elata* (Tian Ma), *Nepeta cataria* (Jing Jie), *Atractylodes japonica* (Cang Shu), *Perilla frutescens* (Zi Su), *Crotonis fructus* (Ba Dou), *Moschus moschiferus* (She Xiang), *Borneolum syntheticum* (Bing Pian), and *Santalum album* L. (Tan Xiang). HFD also contains cinnabar, realgar, and other minerals. As a famous traditional Chinese medicine, it has excellent therapeutic effects on stroke, hemiplegia, epilepsy, mouth-eye crookedness, and other head wind and encephalopathy. It is recorded in “Yi Fang Ju Lei,” “Ying Tong Bai Wen,” etc.

Modern pharmacological studies have shown that HFD has a protective effect on a variety of central nervous system injury and neuroinflammation models. HFD protects mice from bacterial lipopolysaccharide (LPS) plus neurotoxin MPTP toxicity [1] and ameliorates LPS plus pesticide rotenone-induced neuroinflammation and dopaminergic neuron loss in rats [2]. HFD also has modulatory effects on gut microbiota (submitted), which are in line with the clinical experience and theory of traditional Chinese medicines.

Chinese herbal medicine preparations are composed of many different compounds with various structures and functions, and all components act together on multiple targets instead of a specific target to achieve therapeutic effects and to reduce toxicity. Some of the components act as the main medicine (JUN), some as secondary medicine (Zou), some as complementary medicine (Chen), and some as guide-drug (Shi) [3]. HFD is such an example, and the traditional recipe of HFD is more effective than the modified (removing cinnabar and/or realgar) recipe in protecting against LPS-induced neuroinflammation in neuron/microglia cultures [4] and in animals [2]. Thus, to illustrate the antistroke mechanism of HFD more systematically and comprehensively, this research intends to analyze and expound the potential molecular mechanisms of HFD based on system pharmacology. As an emerging discipline, systems pharmacology includes many disciplines such as systems biology, systems pharmacology/toxicology, computational biology, and network analysis, which break the traditional framework (drug-target-disease) to a multilevel network (disease-phenotype-gene-drug) and explore the correlation between drugs and diseases from the perspective of wholeness and systematic view, corresponding to the theory of holistic view and dialectical treatment of traditional Chinese medicine [5, 6].

In this work, the active molecules in HFD were identified that transcend the physiological barriers and interact with the network targets. We aim to use a comprehensive network pharmacology-based approach to investigate the mechanisms of how HFD exerts therapeutic effects on stroke, and the built network was further verified by correlating with the GEO database of antistroke Chinese medicine.

## 2. Materials and Methods

### 2.1. Establishment of a Database of HFD Target Genes and Stroke-Related Genes

Through the Traditional Chinese Medicine Systems Pharmacology (TCMSP) database (https://tcmspw.com/tcmsp.php) [7] and the Traditional Chinese Medicines Integrated Database (TCMID) (http://119.3.41.228:8000/tcmid/) [8], the components and target genes of 13 Chinese herbal medicines of HFD were retrieved from two databases satisfying the criteria of oral bioavailability (OB) greater than or equal to 30% and drug-likeness (DL) greater than or equal to 0.18% [9, 10]. Stroke-related genes were collected through the GeneCards database (https://www.genecards.org/MyGenes/) and the DrugBank database (https://www.drugbank.ca/) using the keyword “Stroke.”

### 2.2. Establishment of Ingredients and Chinese Herbal Medicines Network

Candidate potential ingredients and herbals of HFD were retrieved and screened from TCMSP retrieved and TCMID database. All the ingredients and their quantitative targets were visualized analysis using Cytoscape 3.7.1 software [11].

### 2.3. Intersection Target Constructions of HFD and Stroke

HFD targets and stroke targets were transferred to uniform generic names through the UniProt database (https://www.uniprot.org/). The “Draw Venn Diagram” online tool (http://bioinformatics.psb.ugent.be/webtools/Venn/) was used to input the previously collected HFD genes and stroke genes to achieve common genes [12].

### 2.4. Construction of the Ingredient-Target-Disease Interaction Network of HFD and Stroke

The previously collected active ingredients were combined, and the frequent targets of HFD and stroke were visually analyzed using Cytoscape 3.7.1 software.

### 2.5. Constructions of the Protein-Protein Interaction (PPI) Network Map

The previously collected common targets were entered into the STRING online database (https://string-db.org/), the species were selected as “Homo sapiens,” and the obtained “tsv” file was imported into Cytoscape 3.7.1 software for further analysis of the core network.

### 2.6. Core Network Constructions

In the previously obtained “tsv” file, the top twenty-two targets were selected in the number of nodes, and the “R” 4.0.2 software was run to draw a histogram. Then, the obtained “tsv” file was imported into Cytoscape 3.7.1 software. The MCODE plug-in was run to analyze the core network, and the network ranked first was selected for the next analysis [13].

### 2.7. GO and KEGG Pathway Enrichment Analysis

DAVID online database (https://david.ncifcrf.gov/) was used to perform gene ontology (GO) and Kyoto Encyclopedia of Genes and Genomes (KEGG) pathway enrichment analysis to reflect the biological process, molecular function, cellular component, and pathway of HFD in the treatment of stroke. The results are displayed in a bar chart or bubble chart. The smaller the *p* value is, the higher the degree of enrichment is; the larger the count is, the more genes are enriched on it [14].

### 2.8. Molecular Docking

First, the top 6 core target genes were selected, and the compounds that might regulate these targets were reviewed. The two-dimensional (2D) structure diagrams of these compounds were downloaded through the PubChem database and imported into the Chem3D software to draw three-dimensional structure diagrams and optimize energy and save them in mol2 format. Then, the files were imported into AutoDockTools-1.5.6 software to add the charge and display rotatable keys and then saved in pdbqt format. Next, the protein crystal structures corresponding to the core target genes were downloaded from the PDB database, imported into PyMOL software to remove water molecules and heteromolecules, imported into AutoDockTools-1.5.6 software to add hydrogen atoms, saved to pdbqt format, and imported into Discovery Studio 3.5 Client software to search for active pockets. Finally, the compound is used as a ligand, and the protein corresponding to the core target gene is used as a receptor for molecular docking. PyMOL software and Discovery Studio 3.5 Client were used to analyze and interpret the results.

### 2.9. Correlation with the GEO Database

BaseSpace Correlation Engine (BSCE) (https://www.illumina.com/products/by-type/informatics-products/basespace-correlationengine.html; formerly NextBio) is an RNA sequencing and microarray database curated over 23,000 scientific studies to get data-driven answers for genes, experiments, drugs, and phenotypes for the research. The 26 key targets analyzed by the MCODE plug-in were individually input into BSCE for curated studies, followed by filtering with the keyword “stroke” and then combined as the “template.” Using “Chinese medicine” and “stroke” for curated studies, there is one study using Xuesaitong against MOCA-induced stroke in mice with two biosets and a summary *p* value by the Running Fisher test. The −log(*p* value) was calculated and VLOOKUP with the “template” to make a correlation. This method provides a correlation of the overlapping genes between DEGs and biosets curated in BSCE [15]. Biosets that were positively correlated with the DEGs were predicted to produce similar effects, either directly or indirectly; the larger the −log(*p* value), the higher the degree of similarity. Biosets that were negatively correlated with DEG were predicted to produce opposite effects. The Treeview 1.6 (https://download.cnet.com/TreeView/3000-2352_4-75666005.html) was used to visualize differences [16,17].

**Figure 1:**
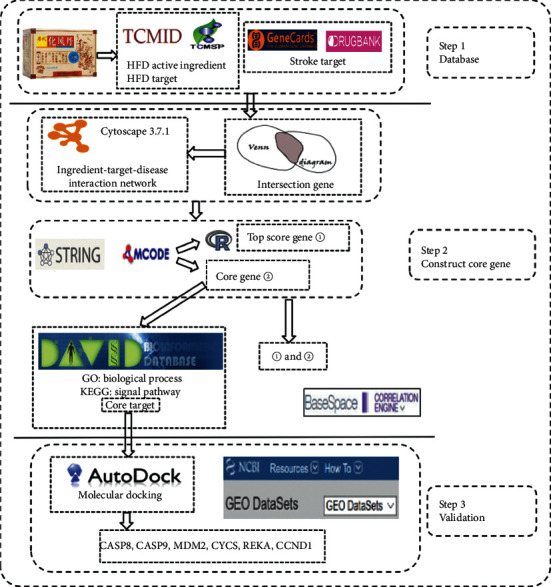
Technological road-map of HFD.

The multistep strategy flow chart in Figure 1 was constructed to explain the method of the manuscript.

## 3. Results and Analysis

### 3.1. HFD Active Ingredients and Chinese Herbal Medicine Network

**Figure 2:**
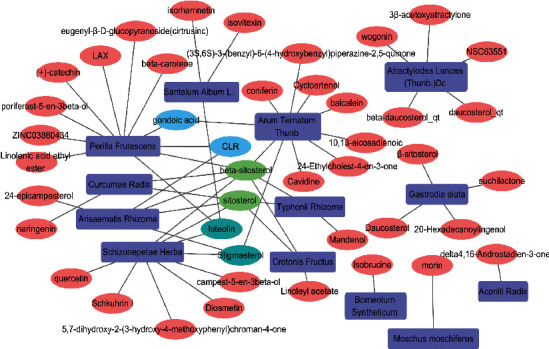
HFD active ingredients and Chinese herbal medicine network.

The 13 Chinese herbal medicines of HFD were searched through the TCMSP and TCMID databases, and there were 42 active ingredients that met the screening conditions (OB ≥ 30%, DL ≥ 0.18), including 3 in *Typhonii rhizoma*, 10 in *Arum ternatum* Thunb, 5 in Aconiti Radix, 3 in Curcumae Radix, 3 in *Crotonis fructus*, 1 in *Borneolum syntheticum*, 5 in *Atractylodes lancea* (Thunb.) Dc, 9 in *Schizonepetae herba*, 1 in *Moschus moschiferus*, 3 in *Santalum album* L., 4 in *Gastrodia elata*, 5 in *Arisaematis rhizoma*, and 11 in *Perilla frutescens* as shown in Figure 2.

### 3.2. Intersection Targets of HFD and Stroke

**Figure 3:**
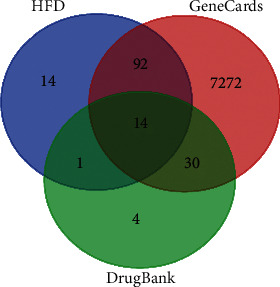
Intersection targets' Venn diagram of HFD and stroke.

According to the 42 active ingredients of HFD, 121 targets were retrieved in the TCMSP database. 7408 and 49 targets were achieved by searching for “Stroke” in GeneCards and DrugBank databases, respectively. The targets obtained above were entered into the Venn database to obtain a common target, as shown in Figure 3. 106 targets were intersected by HFD and GeneCards and 15 targets were intersected by HFD and DrugBank. A total of 107 targets were analyzed for the next step.

### 3.3. Ingredient-Target-Disease Interaction Network of HFD and Stroke

**Figure 4:**
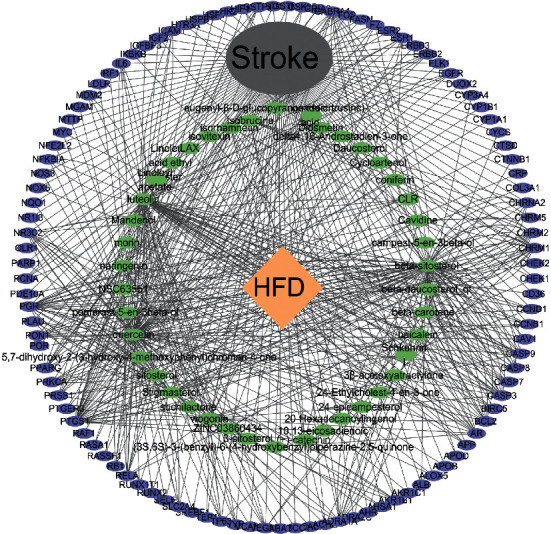
Ingredient-target-disease interaction network of HFD and stroke.

The 42 active ingredients of HFD collected before and 107 intersection genes of HFD and stroke were imported into Cytoscape 3.7.1 software for visual analysis. As shown in Figure 4, green is the active ingredient and purple is the target, showing the active ingredient direct relationship network with target diseases and HFD.

### 3.4. Core Network

**Figure 5:**
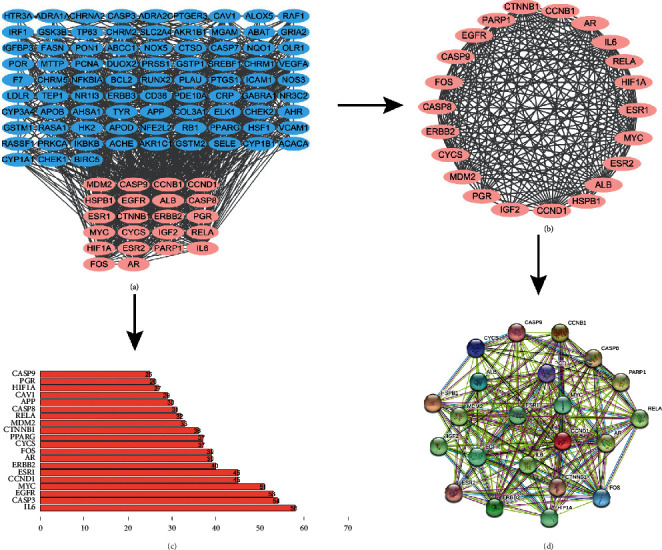
Analysis of core genes at the intersection of HFD regulatory genes and stroke target genes. HFD regulatory gene and stroke target gene intersection (a). Core genes of HFD and stroke (b). Barplot statistical results of the interaction between HFD and stroke regulatory genes (c). PPI network of core gene corresponding protein (d).

The previously collected 107 common targets were entered into the STRING online database, the species were selected as “Homo sapiens,” and the obtained “tsv” file was imported into Cytoscape 3.7.1 software, as shown in Figure 5(a) (one target has not connected with others, which cannot be displayed). The 22 targets in pink are the first-ranked core networks analyzed by the MCODE plug-in. The 22 targets are entered into the Cytoscape 3.7.1 software to show the network relationship, as shown in Figure 5(b). The “tsv” file was used to run the “R” 4.0.2 software to draw a histogram, and the first 20 targets of the number of nodes were selected to display as shown in Figure 5(c).

### 3.5. Constructions of the Protein-Protein Interaction (PPI) Network Map

The 22 core targets selected by the MCODE plug-in were entered into the STRING database for PPI network analysis (Figure 5(d)). There were 22 nodes and 194 edges in the PPI network, and the average node degree is 17.6, the number of the expected edges is 56, the average local clustering coefficient is 0.887, and the PPI enrichment *p* value is <1.0*e* − 16. Besides, in all the nodes in Figure 5, the darker the color is, the more important it was.

### 3.6. GO Analysis and KEGG Pathway Enrichment Analysis

**Figure 6:**
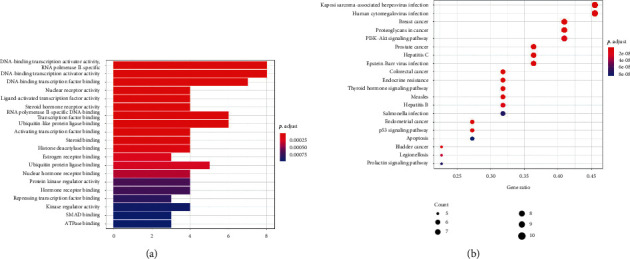
Enrichment analysis. GO analysis of HFD and stroke target genes (a). KEGG analysis of HFD and stroke target genes (b).

R language was used for GO analysis and KEGG analysis. Based on the DAVID database, it was used to analyze the core intersection genes of HFD and stroke. Go analysis includes a biological process (BP), cell composition (CC), and molecular function (MF). Fifty-one significant changes in biological processes were screened, and the top 20 were displayed as bar graphs (Figure 6(a)). KEGG pathway enrichment analysis screened 60 signal pathways with significant enrichment of core genes, of which the top 20 were selected and represented by a bubble chart (see Figure 6(b)).

### 3.7. Verification of the Interaction between Active Ingredients and Target Genes

**Figure 7:**
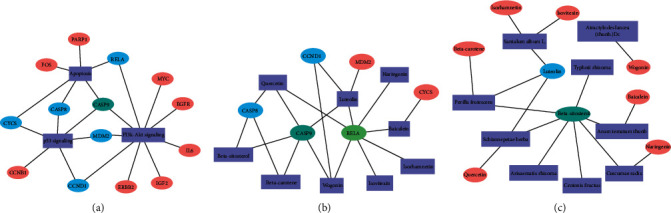
Key signaling pathways and target genes of HFD and stroke intersection genes (a). Key target genes and ingredients of HFD and stroke intersection genes (b). Key ingredients and Herbal medicine of HFD and stroke intersection genes (c).

Through in-depth analysis, the three most important signaling pathways, apoptosis, phosphatidylinositol-3 kinase (PI3K)/AKT, and P53 signaling pathway, were selected. The genes enriched in the abovementioned pathways include 14 genes in the core genes, in which the six genes were in two or more of the pathways (Figure 7(a)). The top 6 core target genes trace ingredients back to 9 (Figure 7(b)), and 9 kinds of herbs contain these ingredients (Figure 7(c)).

**Figure 8:**
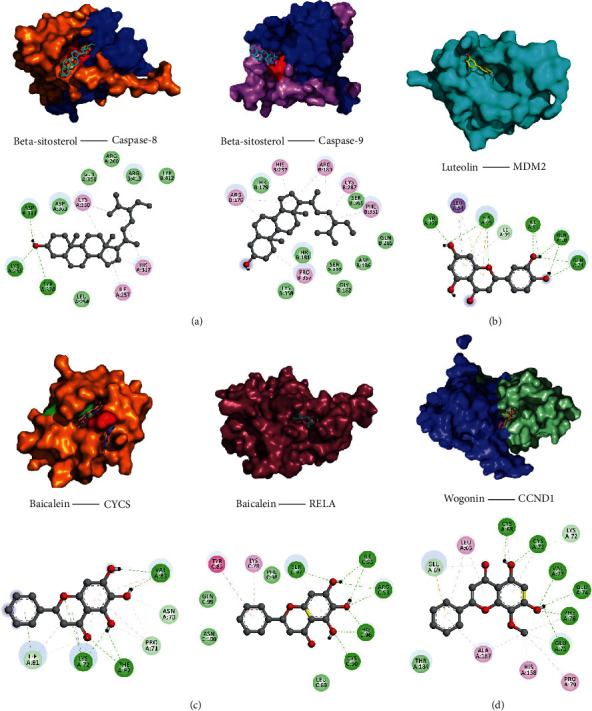
Molecular docking of key target genes and ingredients. Docking concept and binding site analysis of beta-sitosterol (a). Docking concept and binding site analysis of luteolin (b). Docking concept and binding site analysis of baicalein (c). Docking concept and binding site analysis of wogonin (d).

Molecular docking was used for verification of the interaction between active ingredients and target genes. The results obtained by the molecular docking software are shown in Supplementary Table 1. From the results, the lowest binding free energy of beta-sitosterol and caspase-8 was −8.64 kcal/mol. There are Alkyl/Pi-Alkyl hydrophobic interactions between Ile257, His317, Cys360, and beta-sitosterol. Among them, His317 and Cys360 belong to the active site of the caspase-8 protein. In addition, there were three hydrogen bonds: Lys253, Tyr324, Asp319, and beta-sitosterol; there was van der Waals force between Asp363, Gln358, Arg260, Arg413, Tyr412, and beta-sitosterol. On the other hand, the lowest binding free energy of beta-sitosterol and caspase-9 was -9.0 kcal/mol, and there are hydrophobic interactions between His237, Cys287, Arg178, Arg180, Phe351, Pro357, and beta-sitosterol. His237 and Cys287 are the key amino acid residues in the active site of the caspase-9 protein. There are van der Waals interactions between Thr179, Thr181, Lys358, Ser183, Ser361, Asp186, Gln285, Gly182, and beta-sitosterol (Figure 8(a)). The lowest binding free energy of luteolin and MDM2 was −6.97 kcal/mol, there were 9 hydrogen bond forces between Tyr100, His96, Ile19, Gln18, Gln24, and luteolin, and there was a carbon-hydrogen bond between Ile99 and luteolin. In addition, there was a Pi-Cation hydrophobic force between His96 and luteolin, and there is a Pi-Sigma hydrophobic force between Leu54 and luteolin (Figure 8(b)). The lowest binding free energy of baicalein and Cycs was −7.35 kcal/mol, and there were Pi-Sigma and Pi-Alkyl hydrophobic interactions between Ile81, Lys72, Pro71, and baicalein. At the same time, 6 hydrogen bonds are established between baicalein and Lys72, Phe82, and Val83. There are also carbon-hydrogen bonds between Ile81, Asn70, and Pro71and baicalein. The lowest binding free energy of baicalein and Rela was −7.01 kcal/mol, and there were 8 hydrogen bonds between Ser97, Ile95, Arg93, His96, Cys90, and baicalein. In addition, there were Pi-Pi/Pi-Alkyl hydrophobic interactions between Tyr85, Lys78, and baicalein. Gln99, Asn100, Phe98, Leu89, and baicalein had van der Waals forces (Figure 8(c)). The lowest binding free energy of luteolin and CCND1 was −7.35 kcal/mol. Leu65, Ala187, His158, and Pro79 had hydrophobic interactions with luteolin. There were 6 hydrogen bonding forces between Cys68, Cys73, Val77, Glu74, Phe78, Glu75 and luteolin. Glu69, Lys72, Thr184, and luteolin also have van der Waals forces (Figure 8(d)).

### 3.8. Correlation with GEO Database

**Figure 9:**
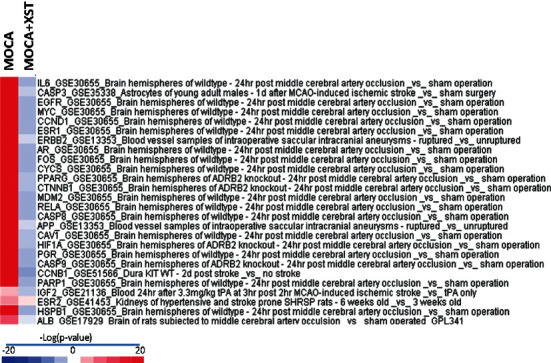
BaseSpace Correlation Engine analysis of 26 target genes with the GSE biosets based on −log (*p* value) with the MOCA biosets (GSE61616). Red indicates the upregulation, and blue indicates the downregulation in the style of Target gene_ GSE_biosets. MOCA bioset (first column) was highly correlated with identified 26 target genes. In MOCA + Xuesaitong treatment (2^nd^ column), all MOCA-induced target changes were returned to the normal or to the opposite direction (negative correlations).

Figure 9 shows 26 core gene targets from Figures 5(b) and 5(c) in correlation with the GEO curated database (keyword: stroke) based on the MOCA stroke model [17] (GSE61616). The selected 26 targets were highly correlated with brain stroke database across curated studies (Figure 9). Xuesaitong treatment reversed all of these changes, indicating that these molecules were valid biomarkers for the therapeutic effects of Chinese medicine against stroke. The correlation of −log(*p*-values) >4 or <−4 with the 26 gene targets in 153 gene biosets (17 GSE studies) except for Igf2 (2.92) was provided as Supplementary Table 2D.

## 4. Discussion

In this study, the active ingredients and potential targets of HFD in the treatment of stroke were studied through network pharmacology. 42 active substances were identified by TCMSP and TCMID (OB ≥ 30%, DL ≥ 0.18); 107 targets were identified with GeneCards and DrugBank. MCODE screened out 22 target genes, STRING constructed 194 Edges in PPI, GO analyzed 51 biological processes, and KEGG enriched 60 significantly related pathways. Molecular docking of 6 targets with 4 active ingredients provided an in-depth analysis of network pharmacology. In addition, the 26 targets from Figures 5(b) and 5(c) were highly correlated with the GEO database, and the antistroke effects of Xuesaitong in the database were verified with these targets. It suggests that the key genes screened in this study may become a potential biomarker for evaluating stroke severity and stroke treatment efficacy of Chinese medicines.

Since its creation in the Ming Dynasty, HFD has played an important role in the improvement of stroke and poststroke symptoms. The formulation of HFD has been continuously improved; for example, through the fermentation, the macromolecules could be degraded into small molecular substances by microorganisms to improve the bioavailability, and the processing by the “Shui-Fei method” is important to reduce the toxicity of heavy metals such as cinnabar and realgar. In recent years, studies have demonstrated that the safety of HFD is different from environmental mercury and arsenic compounds [18–21]. However, the mechanism of HFD has not been fully elucidated.

Due to the complexity of the components of traditional Chinese medicine, it is difficult to fully discuss the mechanism of HFD in the treatment of brain diseases through the point-to-point research model of “the animal model-signal pathway,” which requires a lot of tedious work and away from the theory of traditional medicine. Network pharmacology studies may provide a novel approach by constructing a drug-target-disease network from the perspective of the intersection of drugs and disease regulation, through the analysis of the action network, the representative active ingredients are screened, and the target genes are verified by molecular docking, to reveal the active ingredients and mechanism of HFD action in stroke prevention and treatment.

In this study, the HFD recipe was first screened by TCMSP and TCMID database, and 13 herbs and 42 ingredients were obtained. Subsequently, 107 interactions with GenCard and DrugBank were found. The core network was obtained through Cytoscape analysis, which contained 22 genes. Typically, the core gene is considered a key role, so we have shown up core gene (Figure 5(b)), STRING constructed 194 Edges in PPI, we selected the intersection of the largest gene interactions which are 26 genes (Figures 5(b) and 5(c)), and the genes screened by the two methods are highly similar, suggesting that the analysis results are more accurate.

GO analyzed 51 biological processes, and KEGG enriched 60 significantly related pathways. In the KEGG analysis, the 3 most important signal pathways were selected through in-depth analysis: apoptosis, phosphatidylinositol-3 kinase (PI3K)/AKT, and P53 signaling pathway. Multiple studies have confirmed that apoptosis is initiated in stroke. Caspase-3, caspase-8, and caspase-9 are important members of the caspase family; upon receipt of specific stress, cytochrome c released by mitochondria will combine with procaspase-9/Apaf-1 to activate and cleave caspase-9 [22]. The cleaved caspase-9 further processes other caspase members, initiates the caspase cascade, and then initiates apoptosis [23]. Activated caspase-8 synergistically cleaves and activates the caspase of downstream effector molecules, such as caspase-1, caspase-3, caspase-6, and caspase-7, and amplifies the apoptosis signal [24]. PI3K/Akt signaling pathway participates in various cellular processes, and the activation of the pathway has been revealed to be implicated in the occurrence and development of angiogenesis, which negatively modulates genes that promote thrombogenicity, vascular permeability, and inflammation, and thereby protects vascular function [25]. RelA, one of the nuclear transcription factor *κ*B (NF-*κ*B)/Rel families, plays an important role in inflammation and immune response, which may be a PI3K-AKT regulatory signal, which in turn regulates the apoptosis pathway [26]. On the other hand, multiple studies have confirmed that drugs can improve stroke symptoms by regulating the PI3K/Akt pathway [27]. Studies have also reported that PI3K/Akt regulates cell apoptosis, and activation of the PI3K/Akt pathway after stroke plays a protective role in neuronal apoptosis [25]. Under pathological conditions of stroke, p53 plays an important role in the regulation of apoptosis and cell cycle [28]. The increased level of Cyclin chaperone D (cyclin D) levels affects the process of cells entering the S phase under the regulation of P53 [9]. The degradation of P53 hinders its role in the regulation of apoptosis [29]. MDM2 is the ubiquitin ligase of p53 and plays a central role in regulating the stability of p53. Akt mediates the phosphorylation of MDM2 at Ser166 and Ser186, increasing its interaction with p300, so that MDM2 mediates the ubiquitination and degradation of p53 [30]. Phosphorylation of MDM2 also blocks its binding to p19ARF and increases the degradation of p53 [31].

It can be seen that multiple pathways play an important role in stroke through their interactions. In order to further verify the interaction between the 6 core genes and the active ingredients, the HFD effective ingredients were docked with the target to molecular events against stroke. Normally, the binding free energy is lower than −5.0 kcal/mol, indicating good binding activity between the docking molecule and the target, and the values are lower than −7.0 kcal/mol indicating strong binding activity, which indicates a significant interaction. As shown by the results, except for luteolin and MDM2 (6.97 kcal/mol), the lowest binding free energy between other small molecules and their targets is all lower than −7.0 kcal/mol.

In order to study this core network in evaluating the general applicability of traditional Chinese medicine in the treatment of stroke, the 26 genes from Figures 5(b) and 5(c) with stroke were curated in the GEO database and then compared with the GSE biosets related to curated studies using Chinese medicine against stroke. The -log(*p* value) was used to study the correlation of selected genes. The cutoff of −log(*p* value) is set at ± 4 [15]. Under this criterion, 153 gene biosets from 17 studies in the GEO database were significantly correlated with MOCA-induced stroke [17]. In Figure 9, the GSE database for brain stroke included mice GSE30655 [32], GSE35338 [33], GSE13353 [34], GSE 51566 [35], rat GSE 61616 [17], GSE21136 [36], GSE41453 [37], and GSE 17929 [38] and was used to evaluate the correlation of the built core gene targets with stroke. All 26 core targets were highly correlated with the MOCA stroke model [17], and more interestingly, when treated with antistroke Chinese medicine Xuesaitong, the increased −log (*p* values) were returned to the normal, or to the opposite direction, confirming the therapeutic effects of this Chinese medicine. It should be mentioned that when there is one type of “Chinese medicine” and “stroke,” only this study was curated in the database; and when there is one type of “cinnabar” and “stroke,” the same study appeared. Cinnabar is an active ingredient in Chinese medicines including HFD and An-Gong-Niu-Huang Wan for brain diseases [18], and the effects of cinnabar-containing Chinese medicines against stroke are worth of further verification.

In summary, this study predicted the active ingredients, targets, and signal pathways of HFD treatment stroke through network pharmacology and verified the core ingredients and targets, laying a foundation for elucidating the mechanism of action. It also provides a systematic evaluation of the degree of stroke and the effect of drug treatment. The selected 26 core targets could be valuable biomarkers to evaluate the efficacy of HFD and Chinese medicines against stroke.

## 5. Conclusion

The mechanism of action of HFD in stroke involves multiple compounds, targets, and pathways. HFD could regulate the symptoms of stroke through signaling pathways with core targets. This work provided a bioinformatic method to clarify the antistroke mechanism of HFD, and the identified core targets could serve as a biomarker to study antistroke traditional Chinese medicines including mineral-containing remedies.

## Data Availability

The data used to support the findings of this study are included within the article.

## Supplementary Materials

Supplementary Materials Supplementary Table 1: the results obtained by the molecular docking software. Supplementary Table 2: correlation analysis of 26 core targets with the GEO database.
