# Supplementary material for: Potential Molecular Target Prediction and Docking Verification of Hua-Feng-Dan in Stroke Based on Network Pharmacology
Source: Evid Based Complement Alternat Med. 2020 Oct 28;2020:8872593. doi: 10.1155/2020/8872593 (PMC7641700; doi:10.1155/2020/8872593)
Supplement: Supplementary Materials — Supplementary Table 1: the results obtained by the molecular docking software. Supplementary Table 2: correlation analysis of 26 core targets with the GEO database. [file 8872593.f1.zip › 8872593.f1/Supplementary Table 1.pdf]

Compound	Compound 2D structure	Target and PDB ID	Structure with initial ligand	Grid box siz	Affinity (kcal/ mol)
beta-sitosterol (CAS:83-46-5)	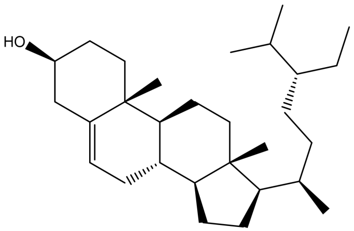	caspase 8 (1QTN)	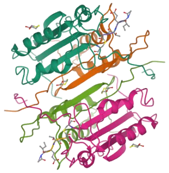	60*60*60	-8.64
		caspase 9 (1NW9)	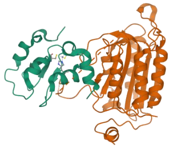	70*80*70	-9.00
Luteolin (CAS： 491-70-3)	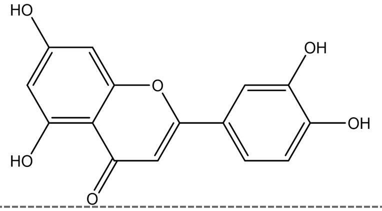	MDM2 (4ZFI)	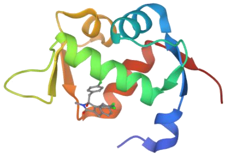	60*60*70	-6.97
Baicalein (CAS: 491-67-8)	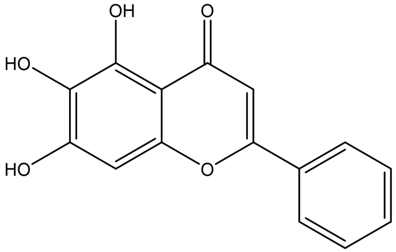	CYCS (3ZCF)	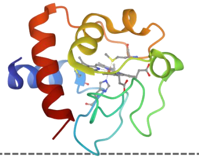	60*60*60	-6.29
		RELA (Homology modeling)	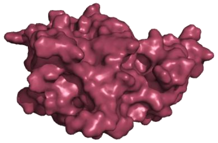	126*126*126	-7.01
Wogonin (CAS： 632-85-9)	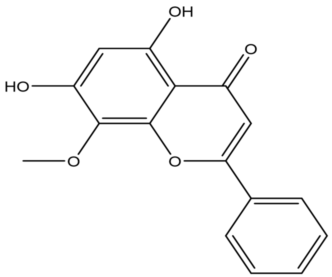	CCND1 (6P8E)	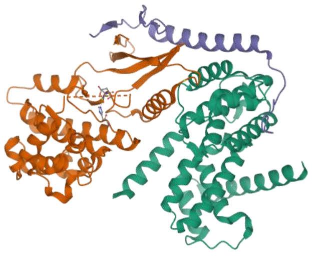	100*100*100	-7.35
