# Supplementary material for: Potential Molecular Target Prediction and Docking Verification of Hua-Feng-Dan in Stroke Based on Network Pharmacology
Source: Evid Based Complement Alternat Med. 2020 Oct 28;2020:8872593. doi: 10.1155/2020/8872593 (PMC7641700; doi:10.1155/2020/8872593)
Supplement: Supplementary Materials — Supplementary Table 1: the results obtained by the molecular docking software. Supplementary Table 2: correlation analysis of 26 core targets with the GEO database. [file 8872593.f1.zip › 8872593.f1/Supplementary Table 2.pdf]

Supplementary Table 2:

Correlation analysis of 26 core targets with GEO database

Genes	Public id	Species	MOCA	XST+MOCA
APP	GSE13353	Human	38.8	-1.52
AR	GSE13353	Human	38.8	-1.52
CASP8	GSE13353	Human	38.8	-1.52
CCNB1	GSE13353	Human	38.8	-1.52
CTNNB1	GSE13353	Human	38.8	-1.52
ERBB2	GSE13353	Human	38.8	-1.52
HIF1A	GSE13353	Human	38.8	-1.52
IL-6	GSE13353	Human	38.8	-1.52
MDM2	GSE13353	Human	38.8	-1.52
MYC	GSE13353	Human	38.8	-1.52
PGR	GSE13353	Human	38.8	-1.52
CASP3	GSE61390	Human	6.39	-2.32
CCNB1	GSE61390	Human	6.39	-2.32
CTNNB1	GSE61390	Human	6.39	-2.32
CYCS	GSE61390	Human	6.39	-2.32
EGFR	GSE61390	Human	6.39	-2.32
MDM2	GSE61390	Human	6.39	-2.32
APP	GSE16561	Human	5.92	-0.485
FOS	GSE16561	Human	5.92	-0.485
HIF1A	GSE16561	Human	5.92	-0.485
MYC	GSE36791	Human	4.7	-0.386
PARP1	GSE36791	Human	4.7	-0.386
HSPB1	GSE118481	Human	4	-0.796
AR	GSE30655	Mice	64.7	-3.7
CASP8	GSE30655	Mice	64.7	-3.7
CAV1	GSE30655	Mice	64.7	-3.7
CCND1	GSE30655	Mice	64.7	-3.7
CYCS	GSE30655	Mice	64.7	-3.7
EGFR	GSE30655	Mice	64.7	-3.7
ESR1	GSE30655	Mice	64.7	-3.7
FOS	GSE30655	Mice	64.7	-3.7
HSPB1	GSE30655	Mice	64.7	-3.7
IL-6	GSE30655	Mice	64.7	-3.7
MDM2	GSE30655	Mice	64.7	-3.7
MYC	GSE30655	Mice	64.7	-3.7
PARP1	GSE30655	Mice	64.7	-3.7
PGR	GSE30655	Mice	64.7	-3.7
RELA	GSE30655	Mice	64.7	-3.7
CASP8	GSE30655	Mice	59.4	-2.72
CASP9	GSE30655	Mice	59.4	-2.72
CAV1	GSE30655	Mice	59.4	-2.72
CCND1	GSE30655	Mice	59.4	-2.72
CTNNB1	GSE30655	Mice	59.4	-2.72
EGFR	GSE30655	Mice	59.4	-2.72
ESR1	GSE30655	Mice	59.4	-2.72
HIF1A	GSE30655	Mice	59.4	-2.72
HSPB1	GSE30655	Mice	59.4	-2.72
IL-6	GSE30655	Mice	59.4	-2.72
MDM2	GSE30655	Mice	59.4	-2.72
MYC	GSE30655	Mice	59.4	-2.72

PARP1	GSE30655	Mice	59.4	-2.72
PPARG	GSE30655	Mice	59.4	-2.72
RELA	GSE30655	Mice	59.4	-2.72
AR	GSE28731	Mice	51.7	-1.54
CASP8	GSE28731	Mice	51.7	-1.54
CASP9	GSE28731	Mice	51.7	-1.54
CCND1	GSE28731	Mice	51.7	-1.54
HIF1A	GSE28731	Mice	51.7	-1.54
HSPB1	GSE28731	Mice	51.7	-1.54
MDM2	GSE28731	Mice	51.7	-1.54
MYC	GSE28731	Mice	51.7	-1.54
RELA	GSE28731	Mice	51.7	-1.54
CCNB1	GSE51566	Mice	45.4	-4.96
CCND1	GSE51566	Mice	45.4	-4.96
MYC	GSE51566	Mice	45.4	-4.96
AR	GSE35338	Mice	22.2	-2.04
CASP3	GSE35338	Mice	22.2	-2.04
CASP8	GSE35338	Mice	22.2	-2.04
CAV1	GSE35338	Mice	22.2	-2.04
CCND1	GSE35338	Mice	22.2	-2.04
CYCS	GSE35338	Mice	22.2	-2.04
EGFR	GSE35338	Mice	22.2	-2.04
HIF1A	GSE35338	Mice	22.2	-2.04
HSPB1	GSE35338	Mice	22.2	-2.04
IL-6	GSE35338	Mice	22.2	-2.04
MDM2	GSE35338	Mice	22.2	-2.04
MYC	GSE35338	Mice	22.2	-2.04
PGR	GSE35338	Mice	22.2	-2.04
RELA	GSE35338	Mice	22.2	-2.04
CYCS	E-MTAB-1	Mice	21.8	-1.68
HIF1A	E-MTAB-1	Mice	21.8	-1.68
IL-6	E-MTAB-1	Mice	21.8	-1.68
APP	GSE35338	Mice	19.2	0
CAV1	GSE35338	Mice	19.2	0
CCNB1	GSE35338	Mice	19.2	0
CCND1	GSE35338	Mice	19.2	0
HSPB1	GSE35338	Mice	19.2	0
IL-6	GSE35338	Mice	19.2	0
CASP8	GSE51566	Mice	18.6	-2.51
CCND1	GSE51566	Mice	18.6	-2.51
CAV1	GSE35338	Mice	15.1	-1.36
CCND1	GSE35338	Mice	15.1	-1.36
EGFR	GSE35338	Mice	15.1	-1.36
HSPB1	GSE35338	Mice	15.1	-1.36
IL-6	GSE35338	Mice	15.1	-1.36
PGR	GSE35338	Mice	15.1	-1.36
CCNB1	GSE51566	Mice	9.17	-2.96
CASP9	GSE28731	Mice	5.39	-0.715
HSPB1	GSE28731	Mice	5.39	-0.715
APP	E-MTAB-1	Mice	-4.47	1.52
CASP3	E-MTAB-1	Mice	-4.47	1.52
ESR1	E-MTAB-1	Mice	-4.47	1.52
CASP3	GSE21393	Mice	-10.4	0
CASP9	GSE21393	Mice	-10.4	0

CCNB1	GSE21393	Mice	-10.4	0
CCND1	GSE21393	Mice	-10.4	0
EGFR	GSE21393	Mice	-10.4	0
ERBB2	GSE21393	Mice	-10.4	0
ESR1	GSE21393	Mice	-10.4	0
ESR1	GSE21393	Mice	-10.4	0
HSPB1	GSE21393	Mice	-10.4	0
PARP1	GSE21393	Mice	-10.4	0
CASP3	GSE51566	Mice	-23.1	2.6
CASP8	GSE51566	Mice	-23.1	2.6
CCNB1	GSE51566	Mice	-23.1	2.6
CCND1	GSE51566	Mice	-23.1	2.6
ESR1	GSE51566	Mice	-23.1	2.6
FOS	GSE51566	Mice	-23.1	2.6
MYC	GSE51566	Mice	-23.1	2.6
MYC	GSE51566	Mice	-29.5	2.01
FOS	GSE21136	Rats	12.9	0.296
MYC	GSE21136	Rats	12.9	0.296
PPARG	GSE21136	Rats	11	-0.801
ALB	GSE17929	Rats	9.64	-0.902
HSPB1	GSE17929	Rats	9.64	-0.902
AR	GSE41453	Rats	5.15	1.15
CASP3	GSE41453	Rats	5.15	1.15
CASP8	GSE41453	Rats	5.15	1.15
CCNB1	GSE41453	Rats	5.15	1.15
CTNNB1	GSE41453	Rats	5.15	1.15
CYCS	GSE41453	Rats	5.15	1.15
EGFR	GSE41453	Rats	5.15	1.15
ERBB2	GSE41453	Rats	5.15	1.15
ESR1	GSE41453	Rats	5.15	1.15
ESR2	GSE41453	Rats	5.15	1.15
HSPB1	GSE41453	Rats	5.15	1.15
IL-6	GSE41453	Rats	5.15	1.15
MDM2	GSE41453	Rats	5.15	1.15
MYC	GSE41453	Rats	5.15	1.15
PARP1	GSE41453	Rats	5.15	1.15
PPARG	GSE41453	Rats	5.15	1.15
IGF2	GSE21136	Rats	2.92	-2.51
HSPB1	GSE4206	Rats	-4.7	0.551
ALB	GSE1548	Rats	-4.74	0
CYCS	GSE1548	Rats	-4.74	0
ESR2	GSE1548	Rats	-4.74	0
PPARG	GSE1548	Rats	-4.74	0
CASP3	GSE41453	Rats	-5.31	0.601
CCNB1	GSE41453	Rats	-5.31	0.601
ESR1	GSE41453	Rats	-5.31	0.601
PGR	GSE41453	Rats	-5.31	0.601
FOS	GSE4206	Rats	-6.05	3.7
APP	GSE61616	Rats	-71.9	0

based on MOCA biosets with -log(p-vale) >4 or <4
